# Delayed Ventricular Repolarization and Sodium Channel Current Modification in a Mouse Model of Rett Syndrome

**DOI:** 10.3390/ijms23105735

**Published:** 2022-05-20

**Authors:** Hongwei Cheng, Ian Charles, Andrew F. James, Ana P. Abdala, Jules C. Hancox

**Affiliations:** School of Physiology, Pharmacology and Neuroscience, University of Bristol, University Walk, Bristol BS8 1TD, UK; hongwei.cheng@bristol.ac.uk (H.C.); ian.charles@bristol.ac.uk (I.C.); a.james@bristol.ac.uk (A.F.J.)

**Keywords:** action potential, eleclazine, GS-6615, I_Na_, I_Na,Late_, MECP2, QT interval, ranolazine, Rett syndrome, RTT, sodium channel

## Abstract

Rett syndrome (RTT) is a severe developmental disorder that is strongly linked to mutations in the *MECP2* gene. RTT has been associated with sudden unexplained death and ECG QT interval prolongation. There are mixed reports regarding QT prolongation in mouse models of RTT, with some evidence that loss of *Mecp2* function enhances cardiac late Na current, I_Na,Late_. The present study was undertaken in order to investigate both ECG and ventricular AP characteristics in the *Mecp2^Null/Y^* male murine RTT model and to interrogate both fast I_Na_ and I_Na,Late_ in myocytes from the model. ECG recordings from 8–10-week-old *Mecp2^Null/Y^* male mice revealed prolongation of the QT and rate corrected QT (QTc) intervals and QRS widening compared to wild-type (WT) controls. Action potentials (APs) from *Mecp2^Null/Y^* myocytes exhibited longer APD_75_ and APD_90_ values, increased triangulation and instability. I_Na,Late_ was also significantly larger in *Mecp2^Null/Y^* than WT myocytes and was insensitive to the Nav1.8 inhibitor A-803467. Selective recordings of fast I_Na_ revealed a decrease in peak current amplitude without significant voltage shifts in activation or inactivation V_0.5_. Fast I_Na_ ‘window current’ was reduced in RTT myocytes; small but significant alterations of inactivation and reactivation time-courses were detected. Effects of two I_Na,Late_ inhibitors, ranolazine and GS-6615 (eleclazine), were investigated. Treatment with 30 µM ranolazine produced similar levels of inhibition of I_Na,Late_ in WT and *Mecp2^Null/Y^* myocytes, but produced ventricular AP prolongation not abbreviation. In contrast, 10 µM GS-6615 both inhibited I_Na,Late_ and shortened ventricular AP duration. The observed changes in I_Na_ and I_Na,Late_ can account for the corresponding ECG changes in this RTT model. GS-6615 merits further investigation as a potential treatment for QT prolongation in RTT.

## 1. Introduction

Rett syndrome (RTT; OMIM #312750) is a severe X-chromosome-linked developmental disorder, first identified in the 1960s [1,2]. It is characterized by arrested development (typically between 6–18 months of age), cognitive and motor skill deficits, microencephaly and seizures [3,4,5]. RTT patients also exhibit autonomic dysfunction, with altered heart rate control and respiratory difficulties (including breath holding and hyperventilation [3,5]). The sporadic “typical” or “classical” form of RTT is strongly associated with mutations to the *MECP2* gene, which encodes the X-linked transcriptional regulator Methyl-Cp-binding protein 2 [4,6,7,8,9]. 95–97% of patients with typical RTT exhibit *MECP2* mutations [3,10]. Patients with RTT are nearly all female; males exhibit more severe cardiac and respiratory abnormalities and the majority die within a year of birth [4]. However, whilst X-linked dominance has long been considered to be characteristic of RTT, *MECP2* mutations have infrequently also been reported in males that exhibit a classical RTT phenotype; such cases are associated with somatic mosaicism or possession of an extra X chromosome [11].

The annual mortality rate in RTT is 1.2%, of which deaths approximately 26% are sudden and unexpected [12]. It is striking that a proportion of patients with typical RTT have abnormal QT intervals and T waves on the electrocardiogram [13,14,15]. Thus, Sekul and colleagues reported prolonged rate corrected QT (QT_c_) intervals and T wave abnormalities in 41% of girls with RTT in comparison to age-matched controls [13]. The proportion of QT_c_ abnormalities and T wave changes increased with increasing severity of the syndrome; the authors suggested a possible link between such changes and sudden, unexpected death in RTT [13]. Ellaway et al. performed electrocardiography and 24-h Holter monitoring of 34 girls with RTT and found a prolonged QT_c_ interval in 9 girls, and an upper borderline value in 10, compared with age-matched healthy controls [14]. Guideri et al. subsequently investigated heart rate variability and QT_c_ intervals in patients with classic and atypical RTT [15]: 55% of girls with classic RTT exhibited QT_c_ interval prolongation, compared to 20% with atypical RTT. More recently, Crosson et al. have reported a lower prevalence of QT_c_ prolongation (7% of a cohort of 100 patients with *MECP2* mutations) [16]. However, whilst in that study patients with a number of *MECP2* mutations demonstrated no QT_c_ prolongation, relatively high proportions of patients (25% in each case) with the R255* mutation or with large deletions exhibited QT_c_ prolongation [16]. A recent study employing serial ECG measurements reported that 12 of 129 patients (9.3%) initially had prolonged QT_c_ intervals at baseline, with 26 patients developing QT_c_ prolongation during follow up (a median time of 1 year and 7 months), with those with the T158M mutation more likely to develop QT_c_ prolongation over time [17].

Murine and primate models of *MECP2*-linked RTT have been reported to exhibit QT_c_ interval prolongation [18,19,20,21]. TALEN-edited *MECP2* mutant cynomolgus monkeys display both a reduction in mean heart rate and increase in QT_c_ interval [21]. *Mecp2* genetically modified mouse models have been widely used in pre-clinical research into the syndrome [4,22]. Restoration of MECP2 function in *Mecp2*-deficient mice is able to reverse neurological deficits in adult animals, consistent with RTT being developmental but not neurodegenerative in nature [23]. The results of experiments on 2–3 month old *Mecp2^Null/Y^* mice have shown these to exhibit increased susceptibility to ventricular arrhythmia and prolongation of the QT_c_ interval [18]. QT_c_ prolongation was found to be linked to an increase in the persistent, or ‘late’ sodium current, I_Na,Late_, in recordings from *Mecp2^Null/Y^* ventricular myocytes under sodium current (I_Na_) selective conditions [18]. The antiseizure drug phenytoin reduced I_Na,Late_ and mitigated QT_c_ prolongation and ventricular arrhythmia susceptibility [18]. Two further subsequent studies of the murine *Mecp2* model have also reported QT_c_ prolongation [19,20]. In one of these, chronic phenytoin administration was found to completely abolish ventricular arrhythmias, but unfortunately also to worsen breathing irregularities in RTT animals [19]. However, a separate study, in which measurements were made at 6 and 8 weeks of age, has failed to observe QT_c_ prolongation in *Mecp2^Null/Y^* mice [24]. To date, only one study has presented direct recordings of I_Na,Late_ from the *Mecp2* null mouse model [18] and no published investigation has incorporated direct recordings of ventricular action potentials (AP) in order to monitor AP repolarization directly. Furthermore, there is little information available on the effects of the model on the rapid sodium current component I_Na_ and its kinetics and the few data that exist were derived under conditions optimised for measurement of I_Na,Late_ [18], which are not best suited for the accurate interrogation of fast I_Na_. The present study was undertaken in order to investigate both ECG and ventricular AP characteristics in the *Mecp2^Null/Y^* male murine model and to interrogate both I_Na_ and I_Na,Late_ in myocytes from the model.

## 2. Results

### 2.1. Delayed Ventricular Repolarization in Mecp2^Null/Y^ Mice

**Figure 1:**
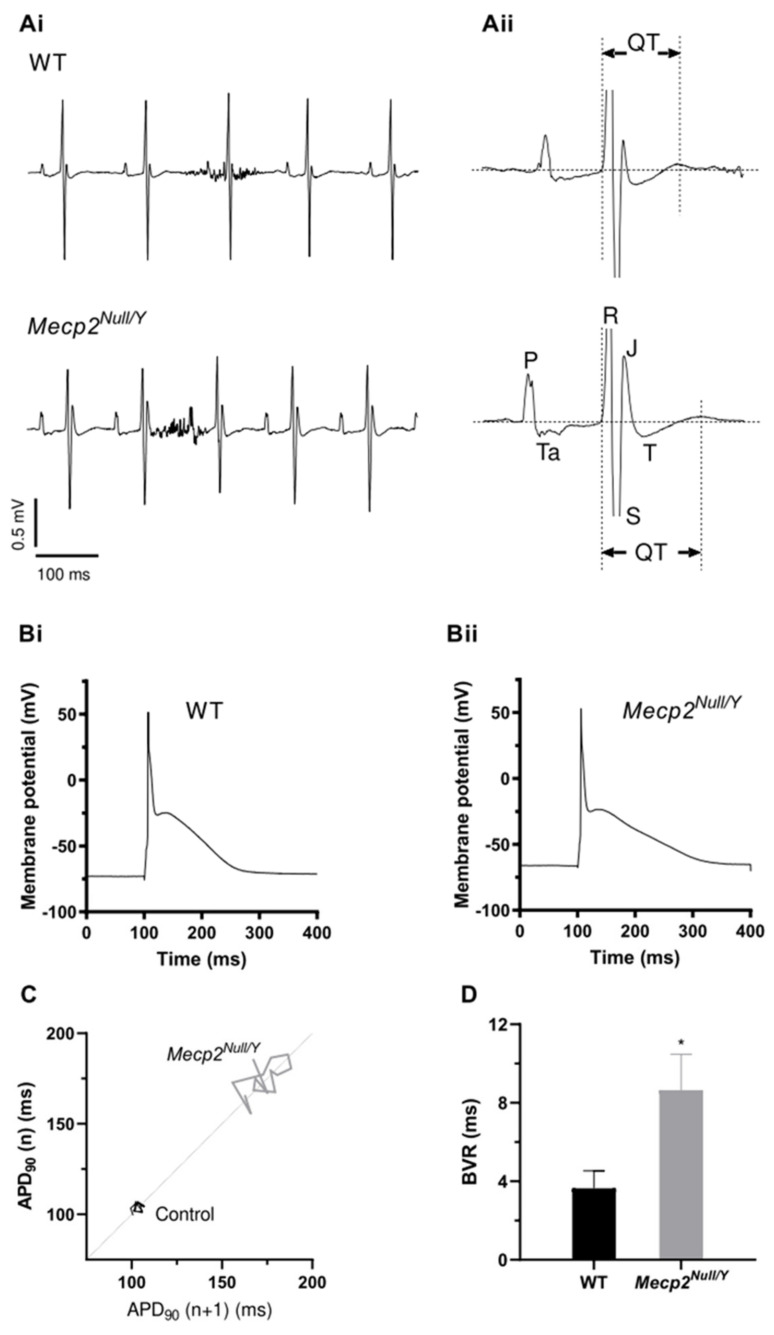
ECG and AP measurements from WT and *Mecp2^Null/Y^* animals. (**A**) Upper and lower panels of (**Ai**) show respectively ECG records from WT and *Mecp2^Null/Y^* mice. The periods of high frequency noise in each trace represent breathing interference. In (**Aii**) an individual expanded ECG cycle is shown for each of WT (upper) and *Mecp2^Null/Y^* (lower) conditions, with labelling of different parts of the ECG complex and the QT intervals shown. (**B**) shows representative recordings of APs (elicited at 1 Hz) from WT (**Bi**) and *Mecp2^Null/Y^* (**Bii**) isolated ventricular myocytes. (**C**) Poincaré plots of APD_90_ from 15 consecutive APs illustrating increased beat-to-beat variability of repolarization (BVR) in *Mecp2^Null/^*^Y^ compared to WT myocytes. (**D**) Mean data showing BVR increase in *Mecp2^Null/Y^* myocytes (n = 23 cells from 12 mice) compared to WT myocytes (n = 23 cells from 9 mice). BVR of APD_90_ was calculated by using equation 3. * denotes *p* < 0.05, unpaired *t* test, unequal variances assumed.

ECG measurements were made from anaesthetised *Mecp2^Null/Y^* mice at 8–10 weeks of age and compared with those from age-matched control male mice. This time range was chosen as it lies within the 2–3 month age range selected by McCauley et al. [18], at which animals exhibit severe motor and behavioural phenotypes. Figure 1(Ai) shows representative ECG traces from WT (upper panel) and *Mecp2^Null/Y^* (lower panel) mice, with the QT interval for a single ECG complex from each strain highlighted in Figure 1(Aii). Table 1 summarizes mean ECG data from 12 WT and 12 *Mecp2^Null/Y^* mice. Heart rate and R–R interval values were similar between WT and mutant strains. However, there was a small but statistically significant increase in QRS interval duration in the RTT model strain (12.2 ± 0.5 ms vs 10.8 ± 0.2 ms in WT; *p* < 0.01). Uncorrected QT interval duration was also significantly greater in RTT (56.9 ± 1.8 ms) than in control (51.9 ± 0.9 ms) mice (*p* < 0.05). We applied two different methods of rate correction to the QT interval ([18,25,26]; see Materials and Methods), which resulted in different absolute QT_c_ values, each of which demonstrated that the mean QT_c_ interval of *Mecp2^Null/Y^* mice was significantly greater (by 5.3–5.6 ms) than that of WT controls (Table 1). Respiratory rate was also monitored during ECG measurement and observed to be significantly slower in RTT than control animals (Table 1). Our ECG measurements are consistent with the original findings of McCauley et al. [18] that *Mecp2^Null/Y^* mice develop prolonged QT intervals.

AP measurements were made at 1 Hz from isolated ventricular myocytes, as described in the Methods. Figure 1(Bi,Bii) show representative APs from WT and *Mecp2^Null/Y^* myocytes respectively, whilst Table 2 summarizes mean AP parameters from 27 myocytes from 9 WT animals and 29 myocytes from 13 *Mecp2^Null/Y^* mice. Resting membrane potential (RMP) was ~2.7 mV less negative in RTT myocytes (*p* < 0.05). The threshold amplitude of the fixed duration (3 ms) depolarizing current stimulus required to elicit APs was significantly reduced in myocytes from RTT animals. However, whilst there was a tendency for both AP overshoot potential and overall AP amplitude to be slightly lower for APs from RTT myocytes these differences did not attain statistical significance. Similarly, mean AP upstroke velocity showed a non-statistically significant trend to be higher in WT than RTT myocytes. AP repolarization parameters were quantified at 10%, 25%, 50%, 75% and 90% of complete repolarization and significant differences were observed for both APD_75_ and APD_90_ (Table 2). Mean APD_90_ was 166.0 ± 10.8 ms and 115.6 ± 9.5 ms in RTT and WT myocytes respectively (*p* < 0.01). AP triangulation was measured as the APD_25_ to APD_90_ difference, which was also shown to be significantly different between WT (113.5 ± 9.4 ms) and RTT (163.5 ± 10.8 ms; *p* < 0.01) myocytes. The Poincaré plot in Figure 1C shows representative beat-to-beat variability (BVR) over 15 successive APs from individual WT and *Mecp2^Null/Y^* myocytes. BVR was quantified using Equation (3) and mean values for WT and *Mecp2^Null/Y^* mice are plotted in Figure 1D: BVR was significantly larger for *Mecp2^Null/Y^* than WT myocytes, indicating increased instability of APD_90_ in myocytes from the RTT model.

**Figure 2:**
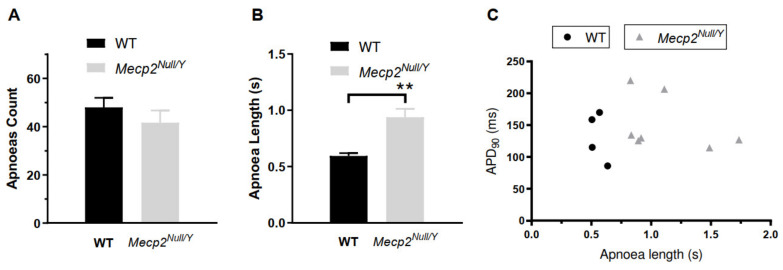
Apnoeas in WT and *Mecp2^Null/Y^* animals. (**A**) shows mean apnoea count from 34 WT and 23 *Mecp2^Null/Y^* animals. These did not differ significantly. An apnoea was counted when the expiration time was longer than four times the average of the expiration time of minute running average. Apnoea was counted for 20 min. (**B**) shows mean apnoea length (duration of each episode) for the same 34 WT and 23 *Mecp2^Null/Y^* animals. ** represents *p* < 0.01; unpaired *t*-test. (**C**) shows a plot of mean ventricular AP duration from 11 mice (4 WT and 7 *Mecp2^Null/Y^*) against apnoea length observed for the same animals. There was no significant correlation between the two parameters (R = −0.07 and *p* = 0.84).

Unrestrained whole body plethysmography was used separately from ECG measurements to monitor occurrence of respiratory apnoea as described previously [27]. In measurements from 34 WT and 23 *Mecp2^Null/Y^* animals, no significant difference was found in numbers of apnoea episodes (Figure 2A, apnoea count). The mean duration of apnoea episodes (Figure 2B, apnoea length) was, however, significantly greater in *Mecp2^Null/Y^* mice. For 11 mice (4 WT and 7 *Mecp2^Null/Y^*) for which ventricular myocytes were isolated following body plethysmography, we plotted mean APD_90_ values against apnoea length (Figure 2C). However, no significant correlation between these two parameters was found (R = −0.07; *p* = 0.84).

### 2.2. Late Sodium Current, I_Na,Late_, Enhancement in Mecp2^Null/Y^ Mice

**Figure 3:**
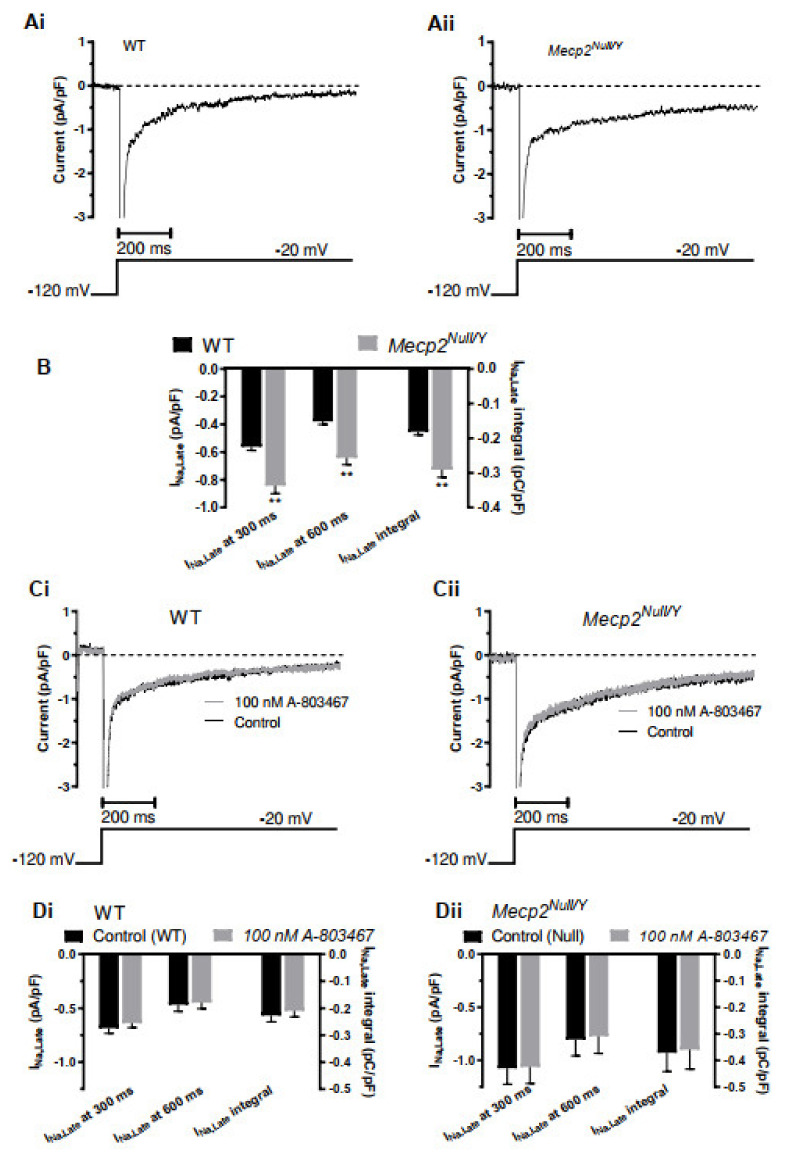
I_Na,Late_ in ventricular myocytes from WT and *Mecp2^Null/Y^* animals. (**A**) shows records of sodium-sensitive (NMDG subtraction) current elicited by depolarization from −120 mV to −20 mV, expanded to focus on the persistent, late current component I_Na,Late_. (**Ai**) shows an example from a WT ventricular myocyte and (**Aii**) shows a corresponding example from a *Mecp2^Null/Y^* myocyte. Voltage protocol is illustrated under current records. (**B**) Bar chart showing comparison between WT and *Mecp2^Null/Y^* I_Na,Late_. Three different measures were taken: I_Na,Late_ density at 300 ms following the start of the applied voltage command; I_Na,Late_ density at 600 ms following the start of the applied voltage command; I_Na,Late_ integral between 350 and 800 ms following the start of the applied voltage command. Data were obtained from 41 myocytes from 20 WT mice and 28 cells from 17 *Mecp2^Null/Y^* mice. ** denotes *p* < 0.01 from comparison with unpaired *t* test assuming unequal variances. (**C**) shows records of sodium-sensitive current elicited by depolarization from −120 mV to −20 mV (protocol shown under currents), expanded to focus on the persistent, late current component I_Na,Late_ to illustrate the effect of the Na_v_1.8 inhibitor A-803467. (**Ci**) shows control and 100 nM A-803467 traces from a WT ventricular myocyte and (**Cii**) shows corresponding records from a similar experiment on a *Mecp2^Null/Y^* myocyte. In each case the control and 100 nM A-803467 traces were closely superimposable. (**D**) mean data for WT (**Di**) and *Mecp2^Null/Y^* myocytes (**Dii**) showing effects of 100 nM A-803467 on each of the 3 measures of I_Na_ introduced in panel B. Data were derived from 8 cells from 6 WT mice and 6 cells from 5 *Mecp2^Null/Y^* mice. None of the measures of I_Na,Late_ showed any significant effect of 100 nM A-803467 (paired *t*-test).

I_Na,Late_ was studied using the recording solutions described in the Methods and a voltage clamp protocol comprised of a 1 s depolarization from −120 to −20 mV (applied at a frequency of 0.1 Hz). With this protocol, the initial rapid I_Na_ component gave way to a small, sustained current component during the applied voltage command. Figure 3(Ai,Aii) respectively show representative records of the Na-sensitive (NMDG-subtraction) current expanded to focus on the sustained component. It is evident that the sustained current, representing I_Na,Late_ is larger for the *Mecp2^Null/Y^* myocyte than the WT example. Three complementary approaches were used to quantify I_Na,Late_, each of which excluded the large initial I_Na_. First, we measured I_Na_ density (pA/pF) at 300 ms into the voltage test command; second, similar measurements were made at a later time-point of 600 ms. The third analysis method was to evaluate the current integral (pC/pF) between 350 and 800 ms into the applied voltage command. Mean data for each of these approaches are plotted in Figure 3B (for 41 cells from 20 WT mice and for 28 cells from 17 *Mecp2^Null/Y^* mice). Each of the three methods used showed a similar result: I_Na,Late_ was significantly augmented in ventricular myocytes from *Mecp2^Null/Y^* mice.

Although cardiac I_Na_ is largely attributable to channels for which the major pore-forming subunit is encoded by *SCN5A* [28] (*scn5a* in mice [29]), the neuronal Na_v_ 1.8 isoform (encoded by *SCN10A*) may also be present and potentially contribute to I_Na,Late_ [30,31]. A-803467 has been shown to be potent and highly selective for Na_v_ 1.8 over Na_v_ 1.5 over a wide range of concentrations (30–1000 nM; [31]). Therefore, we evaluated the effects of 100 nM A-803467 on I_Na,Late_ under our recording conditions. Figure 3(Ci,Cii) show representative traces in the absence and presence of A-803467 for WT and *Mecp2^Null/Y^* myocytes respectively. Traces in the absence and presence of the drug were closely overlain in each case. Figure 3(Di,Dii) respectively show mean data for WT (eight cells from six mice) and *Mecp2^Null/Y^* (six cells from five mice) myocytes. Irrespective of the analysis method used to quantify I_Na,Late_ there was no significant effect of A-803467. As inhibition of Na_v_ 1.8 is near complete at 30 nM [31], this observation precludes Na_v_ 1.8 from contributing significantly to I_Na,Late_ under the conditions of this study.

### 2.3. Alterations to Fast I_Na_ in Mecp2^Null/Y^ Mice

**Figure 4:**
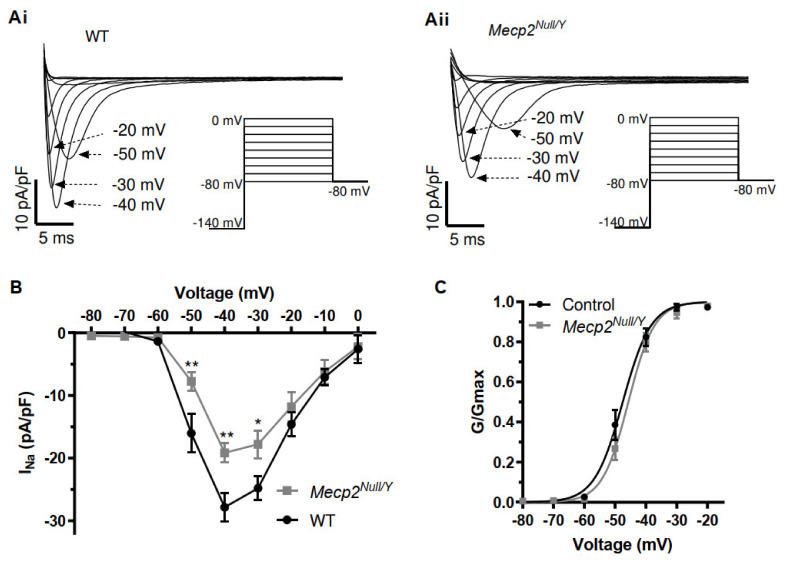
I–V relation and voltage dependent activation of I_Na_. (**A**) Families of I_Na_ from a WT ventricular myocyte (**Ai**), and a *Mecp2^Null/Y^* myocyte (**Aii**). Voltage protocol is shown as insets under current traces. Traces at selected potentials (indicated next to traces) are shown for clarity of display. (**B**) Current–voltage (I–V) relations for peak I_Na_ for WT (n = 11 from 5 mice) and *Mecp2^Null/Y^* myocyte (n = 9 from 5 mice). (**C**) Voltage dependent activation plots for WT and *Mecp2^Null/Y^* myocytes (same experiments as ‘B’). Data were fitted with Equation (5) to yield V_0.5_ and *k* values for WT of −47.4 ± 1.4 mV and 3.6 ± 0.2 mV and for *Mecp2^Null/Y^* of −45.4 ± 1.0 mV and 3.9 ± 0.5 mV. In (**B**) ** denotes *p* < 0.01 and * denotes *p* < 0.05, 2-way repeated measures ANOVA with Bonferroni post-test.

In this study and the earlier investigation from McCauley and colleagues [18], QRS duration differed significantly between WT and *Mecp2^Null/Y^* mice. This observation and the trends towards changes in ventricular AP overshoot/upstroke raise the possibility that differences in fast I_Na_ exist between WT and *Mecp2^Null/Y^* mice. McCauley et al. suggested there is little difference in I_Na_ between the strains, but that was based on measurements from the fast current component under conditions optimised for I_Na,Late_ [18]. The speed and amplitude of I_Na_ make accurate voltage clamp of the current difficult with high [Na^+^]_o_ and cardiac I_Na_ measurement is facilitated by a combination of the use of low [Na^+^]_o_ to reduce I_Na_ amplitude and experimentation at room temperature (e.g., [32,33,34]). Accordingly, measurements of I_Na_ were made at room temperature with pipette and external solutions giving rise to symmetrical 5 mM [Na^+^] (see Methods). Figure 4(Ai,Aii) show families of I_Na_ elicited from WT and *Mecp2^Null/Y^* myocytes, by the protocol shown as insets to these panels. From a holding potential of −80 mV and following a 2 s pre-pulse to −140 mV a series of 250 ms command steps (increasing by 10 mV increments) between −80 and 0 mV were applied (pulse frequency of 0.2 Hz). As shown in Figure 4(Ai,Aii) there was a decrease in current amplitude in the *Mecp2^Null/Y^* condition. Mean current voltage (I-V) relations for I_Na_ from 11 WT myocytes (from 5 mice) and 9 *Mecp2^Null/Y^* myocytes (from 5 mice) are shown in Figure 4B. The I-V plot for *Mecp2^Null/Y^* I_Na_ exhibited statistically significantly decreased I_Na_ density between −50 and −30 mV. I_Na_ conductance values were calculated at each potential for each cell (using Equation (4), Materials and Methods) and G/G_max_ data were then plotted as shown in Figure 4C and fitted with a Boltzmann equation (Equation (5), Materials and Methods) to derive activation V_0.5_ and *k* values. For WT (11 cells from 5 mice) I_Na_, the derived V_0.5_ value was −47.4 ± 1.4 mV and *k* was 3.6 ± 0.2 mV; for *Mecp2^Null/Y^* (9 cells from 5 mice) I_Na_ V_0.5_ was −45.4 ± 1.0 mV and *k* was 3.9 ± 0.5 mV; neither I_Na_ activation parameter differed significantly between the two strains.

**Figure 5:**
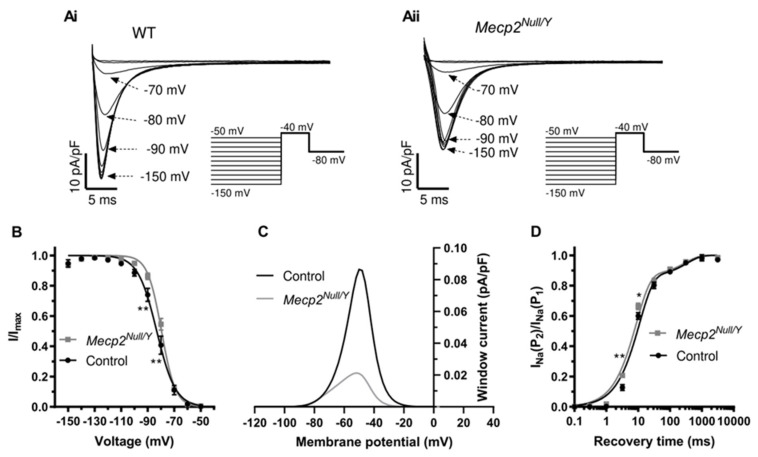
Inactivation properties of I_Na_ and ‘window’ current. (**A**) Families of I_Na_ from a WT ventricular myocyte (**Ai**), and a *Mecp2^Null/Y^* myocyte (**Aii**). Voltage protocol is shown as insets under current traces. Traces elicited during the −40 mV command following the conditioning commands to the voltages indicated next to the current traces. Selected records during the protocol are shown for clarity of display. (**B**) Voltage dependence of I_Na_ inactivation plot in which I/I_max_ represents currents normalized to the maximal I_Na_ amplitude recorded during the −40 mV step, plotted against conditioning voltage. WT data were derived from 9 cells from 5 mice and *Mecp2^Null/Y^* data were derived from 8 cells from 4 mice. Data were fitted with equation 6 to yield inactivation V_0.5_ and *k* values for WT of −83.1 ± 1.7 mV and 6.5 ± 0.3 mV and for *Mecp2^Null/Y^* of −79.6 ± 0.7 mV and 5.1 ± 0.3 mV. ** *p* < 0.01, 2-way repeated measures ANOVA with Bonferroni post-test. (**C**) ‘Window’ I_Na_ calculated at 2 mV intervals, utilizing the product of the mean activation and inactivation variables, G_max_ and driving force at each test potential (see Methods). Window current was decreased for *Mecp2^Null/Y^* compared to WT I_Na_. The negative sign was removed in order to visualize the window in the positive direction. (**D**) Recovery from inactivation (or ‘reactivation’) of I_Na_ obtained using paired pulse protocol described in the Methods/Results text. I_Na_ elicited by a second command at varying inter-pulse intervals was normalized to that elicited by the first pulse and plotted against inter-pulse interval. Mean data from 8 cells from 4 mice for each of WT and *Mecp2^Null/Y^* are plotted. τ_fast_ was 11.0 ± 0.7 ms for WT I_Na_ and 8.6 ± 0.2 ms for *Mecp2^Null/Y^* I_Na_ (*p* < 0.05, unpaired *t* test, unequal variances assumed); τ_Slow_ was 362.0 ± 53.4 ms for WT I_Na_ and 311.5 ± 50.2 ms for *Mecp2^Null/Y^*. The fraction of fast reactivation was 0.85 ± 0.02 for WT and 0.85 ± 0.03 for *Mecp2^Null/Y^*. * *p* < 0.05, ** *p* < 0.01, 2-way repeated measures ANOVA with Bonferroni post-test.

Fast I_Na_ inactivation time-course (τ_inact_) was quantified through exponential fitting of the time-course of current decline of I_Na_ elicited by between −40 and −20 mV (data from experiments shown in Figure 4). At −40 mV, but not −30 or −20 mV, the τ_inact_ was significantly larger for *Mecp2^Null/Y^* than WT I_Na_ (WT and *Mecp2^Null/Y^* τ_inact_ at −40 mV of 2.6 ± 0.2 ms and 3.1 ± 0.1 ms; *p* < 0.05; n = 11 myocytes from 5 mice and n = 9 myocytes from 5 mice for WT and *Mecp2^Null/Y^*, respectively). Voltage dependence of I_Na_ inactivation was evaluated using a paired pulse protocol (shown schematically as insets to Figure 5(Ai,Aii). From a holding potential of −80 mV, 1.5 s conditioning pulses were applied to a range of potentials between −150 and −50 mV in 10 mV increments. Each conditioning pulse was followed by a 40 ms duration test command to −40 mV. The protocol frequency was 0.1 Hz. Figure 5(Ai,Aii) respectively show I_Na_ elicited from WT and *Mecp2^Null/Y^* myocytes during the −40 mV test command, following conditioning pulses to the membrane potential values shown. For each cell studied, the currents during the −40 mV step following the different conditioning potential were normalized to the maximal current observed during the protocol (I/I_max_) and then plotted against conditioning voltage as shown in Figure 5B. Most I/I_max_ values were similar between WT and *Mecp2^Null/Y^* myocytes, although those at −90 and −80 mV were larger in myocytes from the RTT model. Inactivation V_0.5_ and *k* values were obtained from Boltzmann fits to the data with equation 6. For 9 WT cells (from 5 mice) mean V_0.5_ and *k* values were respectively −83.1 ± 1.7 mV and 6.5 ± 0.3 mV. For 8 *Mecp2^Null/Y^* myocytes (from 4 mice), mean V_0.5_ and *k* values were respectively −79.6 ± 0.7 mV (not significantly different from WT) and 5.1 ± 0.3 mV (*p* < 0.01 vs WT). The mean activation and inactivation V_0.5_ and *k* values were then used to calculate the fast I_Na_ “window current” (calculated at 2 mV as the product of activation and inactivation variables, maximal conductance and driving force), which is plotted in Figure 5C. The calculated I_Na_ window was reduced in the *Mecp2^Null/Y^* compared to the WT condition. Consequently, the increased I_Na,Late_ observed in the RTT model cannot be attributed to an increased steady state “window” for fast I_Na_. Nor can the modest depolarization of RMP shown in Table 2 be attributed to increased steady state “window” current.

Recovery of fast I_Na_ from inactivation was determined using a paired pulse protocol in which a 1 s command from a holding potential of –120 mV was applied to −40 mV (P_1_; to activate and then inactivate I_Na_) was followed at varying time-intervals (0.1, 0.3, 1, 3.2, 10, 31.6, 100, 316.2, 1000 and 3162 ms) by a second 40 ms duration command (P_2_) to −40 mV to monitor recovery of I_Na_ amplitude from inactivation (protocol frequency 0.1 Hz). The I_Na_ elicited by P_2_ was expressed as a fraction of that elicited by P_1_ and mean values were plotted against the inter-pulse interval. Recovery from inactivation time-course was quantified through biexponential fitting (Equation (7), Methods). Figure 5D shows mean recovery time-course data, plotted on a logarithmic time-scale (data from eight cells from four mice for each of WT and *Mecp2^Null/Y^* conditions are plotted) [34]. The fast time constant of recovery from inactivation (τ_fast_) was 11.0 ± 0.7 ms for WT I_Na_ and 8.6 ± 0.2 ms for *Mecp2^Null/Y^* I_Na_ (*p* < 0.05), whilst the slow time constant of recovery from inactivation (τ_Slow_) was 362.0 ± 53.4 ms for WT I_Na_ and 311.5 ± 50.2 ms for *Mecp2^Null/Y^* I_Na_ (no significant difference). The proportion of recovery from inactivation fitted by τ_fast_ did not differ significantly between the two strains (0.85 ± 0.02 for WT and 0.85 ± 0.03 for *Mecp2^Null/Y^*). Thus, fast I_Na_ from *Mecp2^Null/Y^* myocytes exhibited slightly faster recovery from inactivation than did WT I_Na_ due to acceleration of the rapid component of I_Na_ reactivation.

### 2.4. Effects of the I_Na,Late_ Inhibitors Ranolazine and Eleclazine (GS-6615)

Ranolazine (Ranexa) was developed as an anti-anginal agent but has undergone extensive investigations for repurposing as an antiarrhythmic drug [35,36,37,38,39]. It is structurally related to lidocaine and is an effective inhibitor of cardiac I_Na,Late_ [40,41].

**Figure 6:**
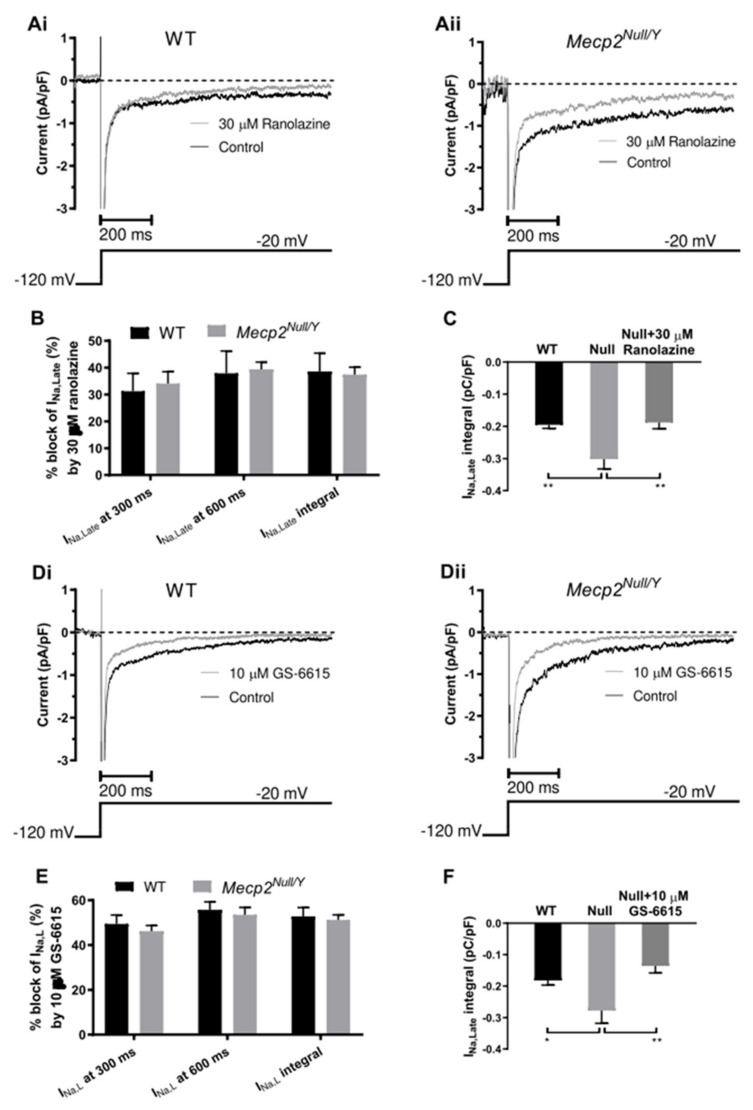
Effects of ranolazine and GS-6615 on WT and *Mecp2^Null/Y^* I_Na,Late_. (**A**) shows records of I_Na,Late_ elicited by depolarization from −120 mV to −20 mV in the absence and presence of 30 µM ranolazine. (**Ai**) shows traces from a WT ventricular myocyte and (**Aii**) shows a corresponding example from a *Mecp2^Null/Y^* myocyte. (**B**) Bar chart showing mean data for WT and *Mecp2^Null/Y^* myocytes showing effects of 30 µM ranolazine on each of the 3 measures of I_Na,Late_ used in Figure 3, expressed as % inhibition of I_Na,Late_. Data were derived from 9 cells from 4 WT mice and 7 cells from 4 *Mecp2^Null/Y^* mice. The extent of inhibition of I_Na,Late_ was similar between WT and *Mecp2^Null/Y^* myocytes. (**C**) Bar charts showing I_Na,Late_ integrals from WT myocytes, *Mecp2^Null/Y^* myocytes and *Mecp2^Null/Y^* myocytes in the presence of ranolazine. Data from 9 cells from 4 WT mice and 7 cells from 4 *Mecp2^Null/Y^* mice are shown. Application of 30 µM ranolazine restored the I_Na,Late_ integral from *Mecp2^Null/Y^* myocytes to a similar value to that found in WT myocytes. (**D**) shows records of I_Na,Late_ elicited by depolarization from −120 mV to −20 mV in the absence and presence of 10 µM GS-6615. (**Di**) shows traces from a WT ventricular myocyte and (**Dii**) shows a corresponding example from a *Mecp2^Null/Y^* myocyte. (**E**) Bar chart showing mean data for WT and *Mecp2^Null/Y^* myocytes showing effects of 10 µM GS-6615 on each of the 3 measures of I_Na,Late_ used in (**B**), expressed as % inhibition of I_Na,Late_. Data were derived from 16 cells from 8 WT mice and 8 cells from 6 *Mecp2^Null/Y^* mice. The extent of inhibition of I_Na,Late_ by GS-6615 was similar between WT and *Mecp2^Null/Y^* myocytes.(**F**) Bar charts showing I_Na,Late_ integrals from WT myocytes, *Mecp2^Null/Y^* myocytes and *Mecp2^Null/Y^* myocytes in the presence of GS-6615. Data from 16 cells from 8 WT mice and 8 cells from 6 *Mecp2^Null/Y^* mice are shown. Application of 10 µM GS-6615 restored the I_Na,Late_ integral from *Mecp2^Null/Y^* myocytes to a value not significantly different from that found in WT myocytes.** denotes *p* < 0.01 and * denotes *p* < 0.05 from one-way ANOVA and Bonferroni post-test.

Figure 6(Ai,Aii) respectively show exemplar traces of the effect of ranolazine (30 µM) on I_Na,Late_ from WT and *Mecp2^Null/Y^* myocytes: in each case the current was reduced following ranolazine exposure. Figure 6B shows the mean the effect of ranolazine evaluated using the three measures described in Figure 3, each plotted as percentage inhibition values (for nine WT myocytes from four mice and seven *Mecp2^Null/Y^* myocytes from four mice). Irrespective of the measurement chosen, ranolazine reduced I_Na,Late_ by a similar percentage in WT and RTT myocytes.

GS-6615 (also known as eleclazine) is a second generation selective I_Na,Late_ inhibitor with an improved structure–activity relationship profile to ranolazine [42] and effectiveness against LQT3 Na channel mutations [43]. Figure 6(Di,Dii) respectively show exemplar traces of the effect of GS-6615 (10 µM) on I_Na,Late_ from WT and *Mecp2^Null/Y^* myocytes. Similar to ranolazine, in each case the current was reduced following GS-6615 exposure. Figure 6E shows the mean effect of GS-6615 evaluated using the three measures used for ranolazine in Figure 6B, each plotted as percentage inhibition values (for 16 WT myocytes from 8 mice and 8 *Mecp2^Null/Y^* myocytes from 6 mice). Irrespective of the measurement chosen, GS-6615 reduced I_Na,Late_ by a similar percentage in WT and RTT myocytes. Figure 6C,F display mean control I_Na,Late_ integral values for WT and *Mecp2^Null/Y^* myocytes together with the *Mecp2^Null/Y^* myocyte integral from the same myocytes following treatment with ranolazine (Figure 6C) and GS-6615 (Figure 6F). Consistent with Figure 3, the *Mecp2^Null/Y^* myocyte I_Na,Late_ integral was significantly larger than that for WT myocytes; however, in the presence of ranolazine or GS-6615 this was no longer the case. Thus, application of 30 µM ranolazine or 10 µM GS-6615 to myocytes from the RTT model restored I_Na,Late_ to levels not significantly different from those in WT myocytes.

**Figure 7:**
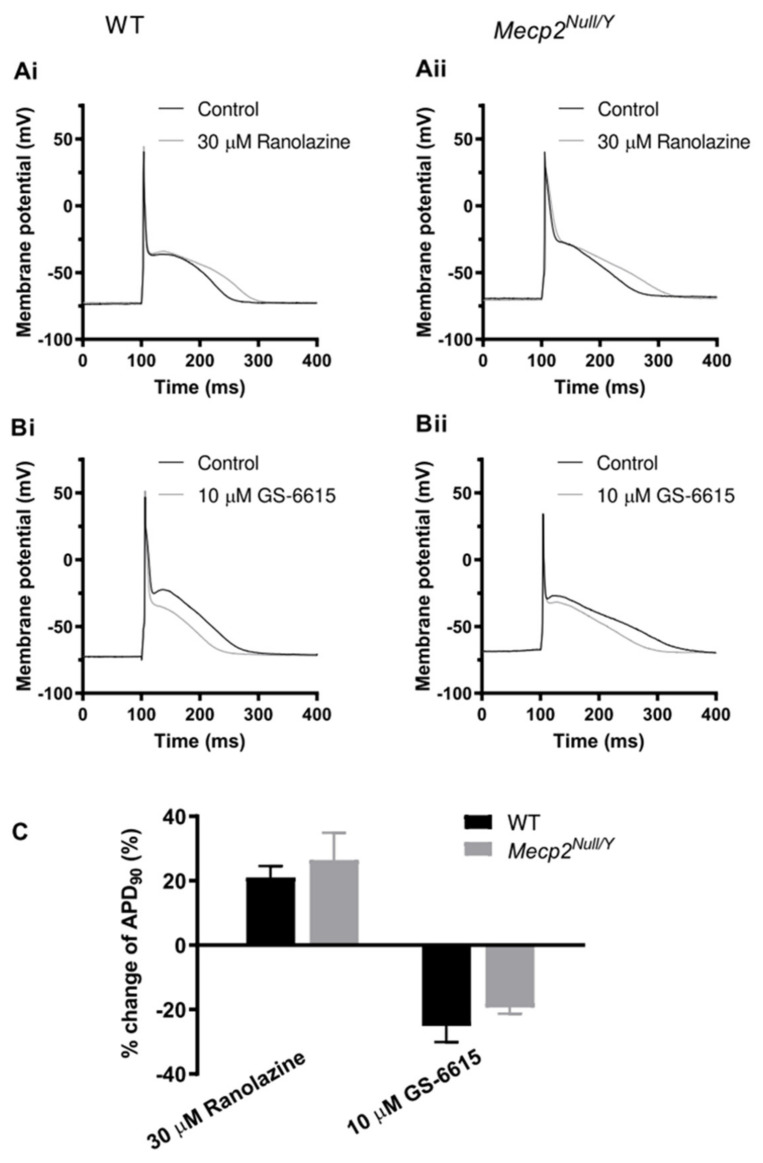
Effect of ranolazine and GS-6615 on ventricular APs. (**A**) Panels Ai and Aii respectively show example APs in control and 30 µM ranolazine for WT (**Ai**) and *Mecp2^Null/Y^* (**Aii**) ventricular myocytes (AP frequency of 1 Hz). (**B**) Panels Bi and Bii respectively show example APs in control and 10 µM GS-6615 for WT (**Bi**) and *Mecp2^Null/Y^* (**Bii**) ventricular myocytes (AP frequency of 1 Hz). (**C**) shows mean data indicating the mean % change in AP duration at 90% repolarization (APD_90_) for each of WT and *Mecp2^Null/Y^* conditions. Plots show data from 7 cells from 4 WT mice and 6 cells from 3 *Mecp2^Null/Y^* mice for the ranolazine groups, and 6 cells from 3 WT mice and 8 cells from 5 *Mecp2^Null/Y^* mice for GS-6615 groups. There was no significant difference between the magnitude of response between WT and *Mecp2^Null/Y^* myocytes for either drug: ranolazine prolonged APD_90_ and GS-6615 abbreviated APD_90_.

In a final series of experiments, effects of ranolazine and GS-6615 on AP duration of WT and *Mecp2^Null/Y^* ventricular myocyte were examined. Figure 7(Ai,Aii) respectively show responses to 30 µM ranolazine of APs from WT (Ai) and *Mecp2^Null/Y^* (Aii) myocytes, whilst Figure 7(Bi,Bii) respectively show effects of 10 µM GS-6615 on APs from WT (Bi) and *Mecp2^Null/Y^* (Bii) myocytes. In each case, GS-6615 led to AP abbreviation, consistent with the compound’s I_Na,Late_ inhibitory action; however, ranolazine lengthened rather than shortened AP duration, as would have been predicted from I_Na,Late_ inhibition. Figure 7C shows the mean percentage changes in APD_90_ produced by each agent, with statistically similar effects on WT and *Mecp2^Null/Y^* myocytes in each case, with APD_90_ lengthening by ranolazine and abbreviation by GS-6615 (the plots show data from seven cells from four WT mice and six cells from three *Mecp2^Null/Y^* mice for the ranolazine groups, and six cells from three WT mice and eight cells from five *Mecp2^Null/Y^* mice for GS-6615 groups). Furthermore, in *Mecp2^Null/Y^* myocytes GS-6615 reduced AP triangulation (from 156.0 ± 14.4 ms to 125.9±9.1 ms; *p* < 0.01). Mean BVR was also reduced by GS-6615, although this difference was not statistically significant (from 8.6 ± 2.8 ms to 5.4 ± 1.4 ms; *p* = 0.09).

## 3. Discussion

### 3.1. Summary of Main Findings

**Figure 8:**
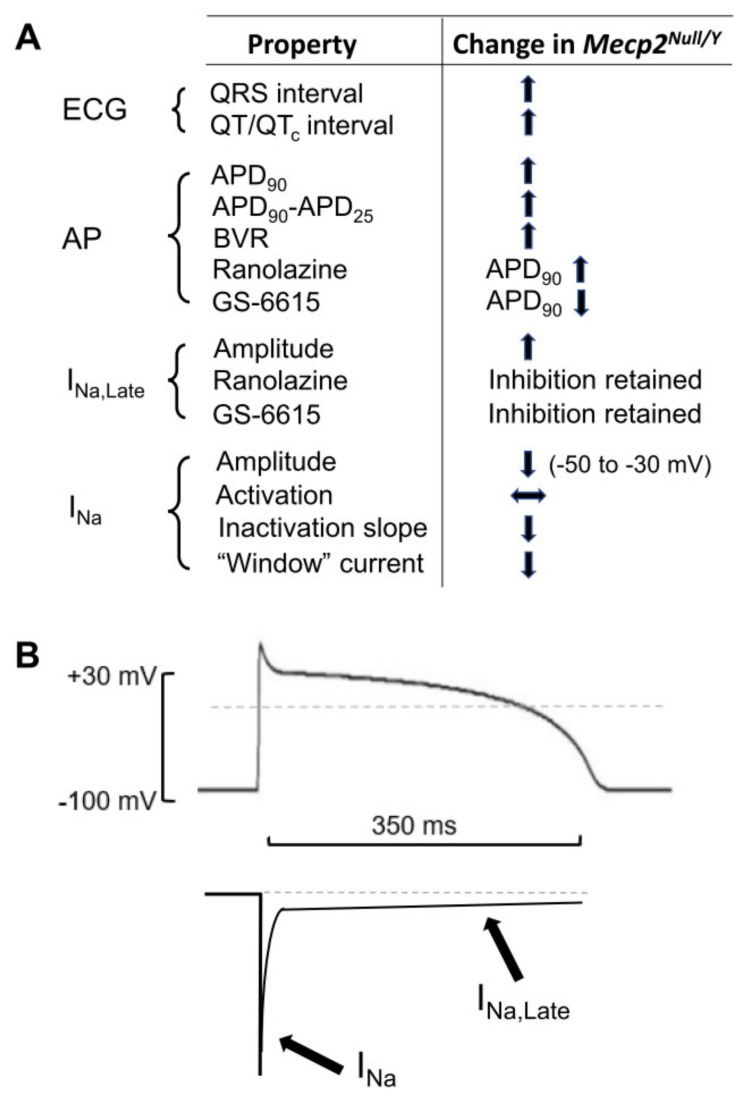
Summary of major findings of this study. (**A**) A schematic showing the changes to the main electrophysiological features studied in this investigation (ECG, action potentials (AP), I_Na,Late_ and I_Na_) in the *Mecp2^Null/Y^* RTT model. The left-hand column itemizes properties studied; the right-hand column shows the direction of alteration in the RTT model compared to WT controls. Upward and downward arrows represent increase and decrease, respectively. The horizontal arrow indicates no change (to I_Na_ activation). For the two drugs studied “inhibition retained” highlights the observation that *Mecp2^Null/Y^* I_Na,Late_ retained the ability to be inhibited by ranolazine and GS-6615. (**B**) Schematic diagram showing relationship between each of I_Na_ and I_Na,Late_ and the ventricular AP (for an AP with a high plateau phase, as occurs in humans). Fast I_Na_ flows during the AP upstroke; I_Na,Late_ is a sustained low amplitude sodium current that flows during the AP plateau phase.

The principal findings of this study are summarized in diagrammatic form in Figure 8A. ECG measurements from *Mecp2^Null/Y^* animals showed statistically significant increases in QRS interval and in both uncorrected QT and rate-corrected QT_c_ intervals, indicative of delayed ventricular repolarization in this model (Figure 8A and Table 1). Repolarization delay was also demonstrated directly through AP measurements from WT and *Mecp2^Null/Y^* ventricular myocytes. Time points up to and including APD_50_ were not significantly different between WT and *Mecp2^Null/Y^* myocytes, but both APD_75_ and APD_90_ were increased (Table 2). In consequence, the difference between APD_25_ and APD_90_, which provides an index of AP triangulation, was significantly increased in RTT myocytes (Table 2 and Figure 8A). Instability of APD_90_, measured as BVR, was also greater in *Mecp2^Null/Y^* myocytes (Figure 1D and Figure 8A). I_Na,Late_ was significantly increased in myocytes from the RTT model (Figure 3 and Figure 8A). In contrast, peak fast I_Na_ amplitude was significantly decreased in *Mecp2^Null/Y^* myocytes without significant change to voltage-dependent activation of the current (Figure 4 and Figure 8A). Comparison of voltage dependent inactivation of I_Na_ from WT and RTT myocytes showed no significant difference in inactivation V_0.5_, but the slope of the inactivation relation was steeper (evidenced by a significant decrease in the slope factor, k; Figure 5B). Consistent with the combination of decreased I_Na_ amplitude over some voltages and a steeper inactivation relation, simulated “window” current was smaller for the *Mecp2^Null/Y^* than WT condition (Figure 5C and Figure 8A). Minor additional changes to I_Na_ were a modest slowing of inactivation time course at −40 mV and a decrease in the fast time constant of recovery from inactivation (Section 2.3; Figure 5D). Our experiments have also shown that I_Na,Late_ from *Mecp2^Null/Y^* myocytes retains sensitivity to inhibition by ranolazine and GS-6615 (Figure 6 and Figure 8A); this was associated with AP abbreviation for GS-6615 but, unexpectedly, with AP prolongation for ranolazine (Figure 7 and Figure 8A).

Figure 8B illustrates, in schematic form, the relationship between each of fast I_Na_ and I_Na,Late_ and the ventricular AP (shown with a high plateau phase as occurs in humans). Fast I_Na_ flows during the AP upstroke, whilst I_Na,Late_ provides sustained inward current during the AP plateau phase. This schematic illustration may aid contextualization of aspects of our findings discussed below.

### 3.2. QT Interval and AP Prolongation

Although the prevalence of QT_c_ interval prolongation in RTT patients varies between studies, it clear is that this ECG change does occur in some patients [13,14,15,16,17,18]. Moreover, the finding that serial ECG measurements within a single cohort have revealed increases during follow up in the number of those exhibiting prolonged QT intervals [17] indicates that a lack of QT_c_ prolongation in a single ECG measurement may not be definitive, and therefore repeated testing over time may be prudent. The results of the present study have shown very similar ECG changes in *Mecp2^Null/Y^* animals to those found in the study of McCauley et al. [18]: QRS widening and QT/QT_c_ interval prolongation. The lack of QT_c_ prolongation in *Mecp2^Null/Y^* mice in the study by Hara et al. [24] is at variance with these findings, but measurements were made between 6 and 8 weeks of age and it is possible that the use of animals from earlier timepoints may have influenced the outcome. Two further studies have reported QT prolongation in RTT mice [19,20] and it is notable that this phenomenon has also been seen in a primate model [21]. An observation of QT_c_ prolongation implies that underlying ventricular APs are also prolonged, but this is the first study to provide an explicit demonstration of delayed ventricular AP repolarization in cardiomyocytes from an experimental RTT model. The results of our experiments showed APD prolongation at measurement timepoints (APD_75_ and APD_90_) beyond APD_50_. The lack of significant AP prolongation at early timepoints during repolarization thus resulted in increased AP triangulation. This is significant because augmented AP triangulation is considered to represent a marker of increased proarrhythmic risk [44]. The larger APD_90_ BVR in *Mecp2^Null/Y^* than WT myocytes is indicative of increased AP instability, which can also act as a marker of proarrhythmic risk [44,45]. Our AP measurements also revealed two unexpected alterations that cannot be detected in ECG measurements: the threshold current required to evoke APs was lower in *Mecp2^Null/Y^* myocytes; there was also a small (<3 mV) depolarization of resting membrane potential. These alterations suggest that differences are likely to exist between WT and *Mecp2^Null/Y^* myocytes in addition to alterations in Na channel function (e.g., these two observations could be explained by a reduction in resting potassium conductance (s)). Whilst it was beyond the intended scope of this study to pursue the underlying ionic basis of those additional changes, future work to that end is warranted.

### 3.3. Changes to Fast I_Na_ and I_Na,Late_

To our knowledge, this is the first study to identify changes to fast I_Na_ in the *Mecp2^Null/Y^* RTT model. A previous study included limited data on fast I_Na_ amplitude at a single test voltage, under recording conditions that were optimised for I_Na,Late_, but not for fast I_Na_ [18] and saw no difference in current amplitude. In contrast, our experiments, performed under conditions that facilitate accurate recording of fast I_Na_ [32,33,34], revealed significant reductions in I_Na_ amplitude over the voltage range that encompassed maximal I_Na_ magnitude, without any significant alteration to voltage dependent activation V_0.5_ and with only a modest effect on the slope of voltage-dependent inactivation (Figure 4 and Figure 5). The reduction in I_Na_ amplitude may help explain QRS complex widening in the *Mecp2^Null/Y^* mouse ECG. The modest delay to I_Na_ inactivation time course may be supplemental to this effect.

However, changes to fast I_Na_ are unable to account for altered I_Na,Late_ in the *Mecp2^Null/Y^* RTT model. The fast sodium channel “window” current, denoted by the area of overlap between steady-state activation and inactivation relations, occurs over a range of membrane potentials within which the probability of availability and activation both exceed zero [46]. Noble and Noble have previously highlighted that whilst the I_Na_ window may contribute to a proportion of I_Na,Late_, it cannot account for all of it; they noted that I_Na,Late_ flows at membrane voltages at which Hodgkin–Huxley models of I_Na_ would predict virtually no current [47]. The experiments in the present study have shown that the I_Na_ window was not increased (rather it was modestly decreased) in *Mecp2^Null/Y^* myocytes, and in consequence this cannot explain the increased I_Na,Late_ in myocytes from the RTT model. Fast I_Na_ and I_Na,Late_ can both be carried by Na_v_1.5 channels [46] and our experiments with A-803467 ruled out a role for Na_v_1.8 in the I_Na,Late_ recorded in this study. Previous single channel investigations of I_Na,Late_ have revealed “scattered” and “burst mode” channel openings [48,49,50] and it is notable that “scattered” and “burst mode” single channel behaviours have both been seen in recordings from recombinant Na_v_1.5 channels [50].

Children with RTT exhibit sympathovagal imbalance, with parasympathetic underactivity and sympathetic overactivity [51]. McCauley et al. reported that QT prolongation and I_Na,Late_ augmentation were observed both in a global *Mecp2^Null/Y^* model and in mice with selective nervous system *Mecp2* knockout [18]. This suggests that the cardiac changes linked to repolarization delay are secondary to neural effects in this RTT model. It is therefore striking that ventricular AP prolongation and changes to I_Na_ and I_Na,Late_ can be measured from isolated ventricular myocytes. This indicates that, whatever the identity of the trigger(s) for these changes in the RTT model, they persist in myocytes that are not subject to acute neural modulation. In both humans and mice with RTT, it is thought that chronic intermittent desaturations and re-oxygenations caused by the respiratory abnormalities induce mitochondrial oxidative stress [52] and it is also known that I_Na.Late_ enhancement can occur in pathological states such as hypoxia and ischaemia, with reactive oxygen species (ROS) activation of CaMKII suggested to mediate current enhancement in such circumstances [46,53]. However, although the RTT mice in this study showed an altered respiration rate and the mean duration of apnoea episodes was significantly greater in *Mecp2^Null/Y^* mice, there was no correlation between APD_90_ values and apnoea length in our experiments. Mucerino and colleagues have reported alterations to the carnitine cycle in this mouse model of RTT, with upregulation of carnitine palmitoyl transferase 1A/B and carnitine acylcarnitine translocase in the hearts of *Mecp2^+/−^* mice [20]. Whether or not resultant changes in fatty acid metabolism could influence I_Na,Late_ is unknown at this time. Consequently, although our experiments have characterized the changes to I_Na_ and I_Na,Late_ in the *Mecp2^Null/Y^* RTT model, the underlying bases for these alterations remain to be elucidated.

### 3.4. Actions of Ranolazine and GS-6615

In the earlier study by McCauley and colleagues, β adrenoceptor blocker administration to this model of RTT did not reduce QT_c_ interval or protect against arrhythmia provoked by programmed electrical stimulation (PES) [18]. By contrast, *Mecp2^Null/Y^* myocyte treatment with the anticonvulsant drug phenytoin decreased I_Na,Late_, whilst injection of *Mecp2^Null/Y^* mice with phenytoin reduced QT_c_ interval duration and ventricular arrhythmia susceptibility [18]. A subsequent study confirmed the beneficial effects of phenytoin *Mecp2^Null/Y^* mice (without any accompanying I_Na.Late_ experiments), but it also found that it worsened abnormal breathing patterns [19]. This compromises the suitability of phenytoin as a treatment for prolonged cardiac repolarization in RTT. Retrospective analysis of patients enrolled in the RTT Syndrome Natural History Study found 10 patients with prolonged QT_c_ intervals that shortened after receiving antiepileptic drugs with Na^+^ channel inhibitory properties [19]. Our data constitute independent direct evidence of augmented I_Na,Late_ in a RTT model and taken together with this earlier work they highlight the utility of I_Na,Late_ inhibition as a potential therapeutic strategy for cases in which QT_c_ prolongation is observed.

Both ranolazine and GS-6615 are of interest as I_Na,Late_-targeting antiarrhythmic agents [35,36,37,38,42,43]; both drugs were observed in our experiments to inhibit I_Na,Late_ in ventricular myocytes from WT and RTT animals and to restore I_Na,Late_ in *Mecp2^Null/Y^* myocytes to control levels. The binding of both ranolazine and GS-6615 to Na_v_1.5 channels is sensitive to mutation of aromatic amino-acid residues that contribute to the local anaesthetic (LA) binding site on the channel [43,54]. The retained sensitivity of I_Na,Late_ from *Mecp2^Null/Y^* myocytes to both agents seen here suggests that the LA binding site is unaffected in this RTT model. The finding of prolongation rather than abbreviation of APs by ranolazine suggests that in addition to I_Na,Late_ inhibition, the drug exerted effect(s) on additional ionic current(s) that influence murine AP repolarization. An independent study has also reported murine ventricular AP prolongation with ranolazine, but did not pursue the underlying reason for this [55]. Ranolazine is known to inhibit the *hERG*-mediated rapid delayed rectifier K^+^ current, I_Kr_ [56,57,58]. However, I_Kr_ does not contribute to murine ventricular repolarization [59] and so cannot account for the observed AP prolongation. Indeed, known differences between the K^+^ currents that mediate human and mouse ventricular repolarization [59] make it difficult to extrapolate pharmacological murine AP prolongation to humans. It is notable, however, that LQT3 patients respond to ranolazine with QT_c_ interval shortening [60] and so it remains possible that in humans and species with a human-like complement of repolarizing currents ranolazine could abbreviate repolarization in the RTT setting.

With its enhanced I_Na,Late_ selectivity and demonstrated effectiveness at inhibiting LQT3 mutant Na channels (e.g., [43]), GS-6615 is a potentially attractive agent for mitigating effects of delayed ventricular repolarization. Its actions are abrogated by mutations (F1760A/Y1767A) at the local anaesthetic binding site on Na_v_ 1.5 [43]. In our experiments it both inhibited I_Na,Late_ and abbreviated ventricular AP duration. APD abbreviation was associated with a reduction in AP triangulation in *Mecp2^Null/Y^* myocytes. These findings highlight a potential for GS-6615 to be used in RTT-associated QT_c_ prolongation. Further work is warranted to determine its ability to inhibit provocation of ventricular arrhythmias in the intact heart of this RTT model.

### 3.5. Limitations and Conclusions

Although most human RTT patients are female with few boys surviving beyond one year of age [4], this study was conducted on male *Mecp2^Null/Y^* mice. Female mice from this strain are heterozygous for *Mecp2* deletion (*Mecp2*^+/−^) and in consequence their clinical phenotype develops rather more slowly than in males: QT_c_ prolongation in *Mecp2*^+/-^ animals was absent at 4 months, but present at 10 months in the study by McCauley and colleagues [18]. A more recent study found that at 11 months some *Mecp2*^+/-^ females display QT_c_ prolongation and others do not [20]. These features make characterization of repolarization and repolarization-linked changes I_Na_/I_Na,Late_ much more challenging and costly in females than in males. The reported similarity between ECG changes in older *Mecp2*^+/-^ and younger *Mecp2^Null/Y^* mice [18], gives confidence in the use of *Mecp2^Null/Y^* mice for studying repolarization changes in the model. Both the study of McCauley et al. and our own have identified changes to QRS interval width as well as QT_c_ interval in the *Mecp2^Null/Y^* model. Our data on fast I_Na_ highlight changes to this in RTT myocytes that may underpin or substantially contribute to QRS changes. However, whilst the earlier study investigated arrhythmias induced by programmed electrical stimulation, neither investigation involved conduction mapping and it would thus be useful in future work to perform such mapping to establish whether alterations to I_Na_ in the model result in altered conduction. Our experiments on GS-6615 and ranolazine focused on acute application to isolated ventricular myocytes. Further work is now required at the intact heart and chronic in vivo exposure levels to establish the ability of GS-6615 to protect against QT_c_-linked arrhythmia susceptibility. In the case of ranolazine, further exploration in a non-murine RTT model with ventricular AP repolarization mechanisms closer to those in humans would be useful to explore further its utility in RTT. While it is important that such limitations are acknowledged, they do not diminish the principal findings of this investigation in relation to ECG and AP changes indicative of delayed repolarization in this model of RTT and the changes to I_Na_, I_Na,Late_ and drug responses that we report. As has been highlighted elsewhere, QT/QT_c_ interval prolongation in RTT is of interest not only due to the association of the syndrome with sudden death, but also because of the use in RTT patients of drugs linked to QT_c_ interval prolongation (serotonin-selective reuptake inhibitors, SSRIs) and a need to monitor QT_c_ prolongation in drug trials [61]. The present study extends information on ionic mechanisms underlying delayed repolarization in RTT. Future work is clearly now needed to elucidate the underlying mechanism for I_Na,Late_ augmentation in this RTT model and to establish the therapeutic value of the potential approaches to mitigating QT_c_ prolongation suggested by our findings.

## 4. Materials and Methods

### 4.1. Mouse Model of RTT Used

All experiments were conducted in accord with UK Home Office legislation and were approved by the University of Bristol Animal Welfare Ethical Review Body (AWERB). Studies were performed on male *Mecp2^Null/Y^* mice with deletions of the third and fourth exons (the so-called “Bird” strain [62]), at between 8 and 10 weeks of age, and on wild-type age-matched male littermates. Mice were genotyped using a standard protocol (P Protocol 24870: Standard PCR Assay-Mecp2<tm1.1Bird> Version 6.2). https://www.jax.org/Protocol?stockNumber=003890&protocolID=24870 (accessed/link confirmed live on 17 April 2022). The following primers were used: common-AAA TTG GGT TAC ACC GCT GA; mutant reverse-CCA CCT AGC CTG CCT GTA CT; wild-type reverse-CTG TAT CCT TGG GTC AAG CTG.

### 4.2. Electrocardiogram (ECG) Measurement

Mice were anaesthetized by 1.5% isoflurane and ECG measurements were obtained 5 min after anaesthesia had been established. Surface lead II ECG measurements were made using subcutaneous needle electrodes, with recording using an A-M Systems (Sequim, WA, USA) differential AC amplifier model 1700 and an CED (Cambridge, UK) Micro 1401-3 AD/DA converter. Signals were high-pass filtered at 10 Hz, with a low-pass setting of 1 kHz. The following ECG parameters were analysed: RR interval (and from this heart rate); PR interval; QRS interval; QT interval (and from this, rate-corrected QT (QT_c_) interval); respiration rate (breathing was evident as brief periods of high frequency interference on the ECG recording). Mean values for each ECG parameter for each mouse were obtained from 5 consecutive ECG complexes, avoiding complexes upon which breathing noise was superimposed. The QT interval duration was determined as the interval between onset of the QRS complex and time point after the T-wave peak [18]. QT_c_ interval values were obtained using two distinct rate correction methods (for Equation (1) [25,26]; for Equation (2) [18]):

QT_c_ = QT/(RR/100)^0.5^ (1)

QT_c_ = QT + 0.3173(170-RR) (2)

### 4.3. Unrestrained Whole Body Plethysmography

Respiratory patterns were monitored using unrestrained whole-body plethysmography (Emka Technologies, Paris, France). Unanaesthetised individual mice were placed in a mouse chamber (396 mL) with a bias flow of 0.7–0.8 L/min. Chamber pressure, temperature and humidity were measured to allow accurate calculation of respiratory flow. Mice were allowed to adapt for the first 20 min, and data was analysed in the subsequent 20 min of recording. Analysis was automated and manually checked by an experienced researcher. Timeseries respiratory flow data was analysed using published custom written ‘algorhythms’ in Spike 2 (V8.22, Cambridge Electronic Design, Cambridge, UK) [63]. A running average of the total expiration time for each breath was taken every minute. If an expiration time was longer than 4 times this average, it was counted as an apnoea. Both apnoea count (number of episodes in 20 min) and length (duration of each episode) were recorded. Comparisons were made between *Mecp2^Null/Y^* and WT control mice.

### 4.4. Ventricular Myocyte Isolation

Animals were killed by cervical dislocation, and the heart was then rapidly excised and placed in ice-cold isolation solution supplemented with 0.1 mM CaCl_2_ and 10 U/mL heparin. The isolation solution contained (in mM) 130 NaCl, 5.4 KCl, 0.4 NaH_2_PO_4_, 4.2 HEPES, 10 glucose, 1.4 MgCl_2_, 20 taurine, and 10 creatine (pH 7.4 with NaOH) [64]. The heart was Langendorff perfused at 37 ^o^C at constant pressure of gravity (~80–100 cm H_2_O) with isolation solution for 3 min followed by enzyme solution (isolation solution plus 0.1 mM CaCl_2_, 0.07 mg/mL protease (Sigma, Type XIV), and 0.7 mg/mL collagenase (Worthington, Type 1)) for 15 min. The ventricles were removed and shaken in enzyme solution for 5 min before being filtered and centrifuged. Cells were resuspended in isolation solution plus 0.1 mM CaCl_2_ and stored at room temperature for using within 10 h.

### 4.5. Cellular Electrophysiology

Ventricular myocytes were placed in an experimental chamber mounted on an inverted microscope (Nikon Eclipse TE2000-U) and were superfused with a Tyrode’s solution containing (in mM): 140 NaCl, 4 KCl, 1.5 CaCl_2_, 1 MgCl_2_, 10 glucose, 5 HEPES (pH 7.4 with NaOH). During experimental recordings, solutions were applied to the cell under investigation using a home-built device that was able to exchange local superfusate within 1 s [65]. Patch pipettes were made from borosilicate glass (A-M Systems Inc, Sequim, WA, USA) pulled and fire polished to resistances of 2–3 MΩ (PP-830 and MF83, Narishige, Tokyo, Japan). For whole cell current recording, series resistance values were typically compensated by >70%. Protocols were generated and data recorded online with pClamp 10 and a Digidata 1440A interface (Molecular Devices, San Jose, CA, USA). The digitization rate was 10 kHz. Further details of specific electrophysiological experiments are given below.

### 4.6. Action Potential (AP) Measurement

APs were elicited from single ventricular myocytes placed in the recording chamber of an inverted microscope by brief (3 ms) depolarizing current injection in whole cell patch clamp recordings (membrane potential recording mode) using a stimulus application frequency of 1 Hz. The threshold amplitude for these current pulses is given in Results Table 2. For these experiments, ventricular myocyte preparations were superfused with an external solution containing (in mM): 140 NaCl, 4 KCl, 1.5 CaCl_2_, 1 MgCl_2_, 10 glucose, 5 HEPES (pH 7.4 with NaOH). The pipette solution for these experiments contained (in mM): 110 KCl, 10 NaCl, 0.4 MgCl_2_, 10 HEPES, 5 glucose, 5 K_2_ATP, 0.5 GTP-Tris (pH 7.1 with KOH). AP recordings were made at 35–37 °C. Beat-to-beat variability of AP repolarization (BVR) was quantified at 90% of AP repolarization (APD_90_) for 10–15 consecutive action potentials, as:

BVR = ∑(|APD_90_(n + 1) − APD_90_(n)|)/(n beats × √2) (3)

### 4.7. I_Na,Late_ Measurement

For I_Na,Late_ recordings, pipettes were filled with a solution containing (in mM): 130 CsCl, 10 NaCl, 10 HEPES, 10 EGTA (pH 7.4 with CsOH). Control superfusate contained (in mM): 130 NaCl, 10 CsCl, 1 MgCl_2_, 10 HEPES, 10 Glucose, 1 4-AP (pH 7.4 with NaOH). To obtain I_Na,Late_ as Na^+^_o_ sensitive current, external Na^+^ was replaced with equimolar N-methyl-D-glucamine (NMDG; pH 7.4 with HCl). I_Na,Late_ was elicited by a voltage protocol comprised of a 1 s voltage command to -20 mV from a holding potential of –120 mV (pulse frequency of 0.1 Hz) and measurements were made at room temperature (cf [18]).

### 4.8. I_Na_ Measurement

Fast I_Na_ recordings were made using symmetrical (5 mM) [Na^+^] [66]. The external solution contained (in mM): 140 CsCl, 5 NaCl, 10 Glucose, 10 HEPES, 1 CoCl_2_, 1 MgCl_2_, 1 CaCl_2_ (pH 7.3 with CsOH). The pipette solution for these experiments contained (in mM): 110 CsF, 20 CsCl, 5 NaCl, 5 HEPES, 5 EGTA, 1 MgCl_2_, 5 Mg-ATP, pH 7.2 with CsOH [66]. Fast I_Na_ measurements were made at room temperature. The specific voltage protocols used to characterize I_Na_ are described in the Results section. The data were analysed used the equations below.

In order to quantify the voltage dependence of activation, I_Na_ amplitude data obtained from current voltage (I–V) protocols (see Figure 4, Results) were used to obtain conductance (G) values:

G = I_Na_/(V_m_ − E_rev_) (4)

where V_m_ is the command voltage potential and E_rev_ is the reversal potential extrapolated from the ascending limb of the I_Na_ I–V relation.

Activation relations for I_Na_ were obtained by plotting G/G_max_ (where G_max_ is the maximum value of G obtained during the I–V protocol) against V_m_ values across the range from −80 mV to −20 mV. The resultant plots were then fitted with a Boltzmann equation of the form:

G/G_max_ = 1/[1 + exp((V_0.5_ − V_m_)/*k*)] (5)

where *V*_0.5_ = half-maximal activation voltage and *k* = slope factor for the fitted relation.

To quantify voltage-dependent inactivation of I_Na_, the following equation was used:

I/I_Max_ = 1 − (1/[1 + exp((V_0.5_ − V_m_)/*k*)]) (6)

where I *=* measured I_Na_ magnitude during a command to −40 mV following a test pulse to voltage V_m_ (Figure 5, Results). I_max_ = maximal I_Na_ observed during the −40 mV command during the protocol, V_0.5_ = half-maximal inactivation voltage and *k* = slope factor for the fitted relationship, between −150 and −50 mV.

The I_Na_ window was obtained by using the experimentally derived activation and inactivation V_0.5_ and *k* values and equations 4 and 5 to calculate activation and inactivation variables at 2 mV intervals between −120 mV and +20 mV. The product of activation and inactivation variables was then multiplied by the experimentally obtained G_max_ and driving force at each test voltage. The resulting plots represented the I_Na_ window under WT and RTT conditions.

In order to evaluate recovery of I_Na_ from inactivation, a paired pulse protocol was used in which two depolarizing commands from −120 mV to −40 mV were applied. The first (1 s duration) pulse (P_1_) activated then inactivated I_Na_. The second voltage command (to −40 mV for 40 ms; P_2_) was separated from the first by interpulse intervals of differing durations (0.1, 0.3, 1, 3.2, 10, 31.6, 100, 316.2, 1000, 3162 ms). The data were fitted with the following equation:

I_Na_(P_2_/P_1_) (t) = 1 − (A_f_exp(−t/τ_fast_) + A_s_exp(−t/τ_slow_)) (7)

where I_Na_(P_2_/P_1_) is the ratio of I_Na_ elicited by P_2_ applied after a time interval ‘t’ following P_1_. A_f_ represents the proportion of recovery fitted by a fast time constant τ_fast_ and A_s_ represents the proportion of recovery fitted by a fast time constant τ_slow_.

### 4.9. Data Analysis and Statistics

Data are presented as mean ± SEM. The numbers of myocytes and animals from which particular datasets were derived are given in the relevant Results text and figure legends. Statistical analysis and fits to datasets were performed using Microsoft Excel (Microsoft Corporation, USA), Prism 8.4.3 (Graphpad Software Inc., San Diego, CA, USA) Origin 7.0 (OriginLab, Northampton, MA, USA), and Clampfit of pClamp 10.7 (Molecular Devices, San Jose, CA, USA). Statistical comparisons were made using paired *t*-test, unpaired *t*-test, one-way or two-way ANOVA followed by a Tukey/Bonferroni post-test, with equal or unequal variances as appropriate. *p* < 0.05 was taken as statistically significant.

### 4.10. Drugs

Ranolazine dihydrochloride was obtained from Sequoia Research products Ltd., and 30 mM stock solution was made in distilled water. GS-6615 (also known as eleclazine) was obtained from SYNthesis Med Chem, and 10 mM stock solution was made in DMSO. A-803467 was obtained from Sigma-Aldrich, and 1 mM stock solution was made in DMSO. All stock solutions were stored at −20 °C. Stock solutions were diluted with the external solutions to obtain the final concentrations as given in the Results.

## Figures and Tables

**Table 1:** ECG characteristics and respiration rate.

Parameter	WT	*Mecp2^Null/Y^*	*t*-Test *p* Value
RR (ms)	130.7 ± 2.4	125.8 ± 5.3	0.4132
HR (bpm)	460.8 ± 8.6	485.8 ± 19.3	0.2569
PR (ms)	38.4 ± 0.8	36.4 ± 1.0	0.1271
QRS (ms)	10.8 ± 0.2	12.2 ± 0.5	0.0094
QT (ms)	51.9 ± 0.9	56.9 ± 1.8	0.0233
QT_c_ (ms; Equation (1))	45.5 ± 0.9	50.8 ± 1.0	0.0006
QT_c_ (ms; Equation (2))	64.4 ± 1.1	71.0 ± 1.1	0.0005
Respiratory rate (bpm)	136.7 ± 4.7	104.3 ± 8.2	0.0029

Mean ± SEM ECG parameters and respiration rate for 12 wild-type (WT) and 12 *Mecp2^Null/Y^* mice. Statistical comparisons were made using unpaired *t*-test, assuming unequal or equal variances as appropriate.

**Table 2:** Ventricular action potential (AP) parameters.

Parameter	WT	*Mecp2^Null/Y^*
Resting potential (mV)	72.2 ± 0.8	−69.5 ± 0.9 *
Overshoot (mV)	48.0 ± 2.0	45.4 ± 2.2
Amplitude (mV)	120.2 ± 2.4	115.0 ± 2.8
V_max_ (V s^−1^)	139.8 ± 6.1	128.1 ± 7.7
APD_10_ (ms)	0.6 ± 0.1	0.8 ± 0.2
APD_25_ (ms)	2.1 ± 0.4	2.5 ± 0.3
APD_50_ (ms)	6.7 ± 1.1	8.1 ± 0.9
APD_75_ (ms)	71.1 ± 8.2	99.6 ± 7.9 *
APD_90_ (ms)	115.6 ± 9.5	166.0 ± 10.8 **
APD_90_-APD_25_ (ms)	113.5 ± 9.4	163.5 ± 10.8 **
Threshold stimulus (pA)	907.4 ± 40.9	597.4 ± 29.3 **

Mean ± SEM values for AP parameters for APs recorded from isolated ventricular myocytes: 27 cells from 9 WT mice and 29 cells from 13 *Mecp2^Null/Y^* mice. APs were elicited by 3 ms duration depolarizing current pulses. Threshold values are included in the table. * denotes *p* < 0.05 and ** denotes *p* < 0.01 from unpaired *t*-test, assuming equal or unequal variances as appropriate.

## Data Availability

Data are available on reasonable request to the corresponding authors.
